# Using peer-assisted learning to teach basic surgical skills: medical students’ experiences

**DOI:** 10.3402/meo.v18i0.21065

**Published:** 2013-08-22

**Authors:** Mahdi Saleh, Yashashwi Sinha, Daniel Weinberg

**Affiliations:** School of Medicine, Keele University, Stoke-on-Trent, UK

**Keywords:** medical curriculum, peer-assisted learning, surgical skills, suturing, interrupted suture, medical students

## Abstract

Standard medical curricula in the United Kingdom (UK) typically provide basic surgical-skills teaching before medical students are introduced into the clinical environment. However, these sessions are often led by clinical teaching fellows and/or consultants. Depending on the roles undertaken (e.g., session organizers, peer tutors), a peer-assisted learning (PAL) approach may afford many benefits to teaching surgical skills. At the University of Keele's School of Medicine, informal PAL is used by the Surgical Society to teach basic surgical skills to pre-clinical students. As medical students who assumed different roles within this peer-assisted model, we present our experiences and discuss the possible implications of incorporating such sessions into UK medical curricula. Our anecdotal evidence suggests that a combination of PAL sessions – used as an adjunct to faculty-led sessions – may provide optimal learning opportunities in delivering a basic surgical skills session for pre-clinical students.

Peer-assisted learning (PAL) – the concept of teaching sessions organized by students for students (1) – is a well-established learning tool used in many institutions. Indeed, within the literature, it is sometimes regarded as the quintessential method of learning and reviewing new material effectively and efficiently (2). Within UK medical schools, in particular, this method has been implemented informally through teaching societies run by medical students. The Keele Surgical Society (KSS) at the University of Keele's School of Medicine serves as an example, using a PAL system to teach the basic surgical skills of suturing and knot-tying skills to pre-clinical (first 2 years of medical school) students.

Although suturing workshops are widely offered within most standard UK medical curricula, they are often taught by staff members composed of clinical teaching fellows and/or consultants. In contrast, the benefits of PAL have been well documented: preparing peer tutors for future responsibilities as medical educators; relieving the burden of faculty teaching; and providing a foundation for student relationships that can continue throughout medical school (3). Additionally, studies have shown that students find it less intimidating and display higher levels of enthusiasm in PAL sessions when compared to the traditional faculty-led approach (4).

Currently, sessions of approximately 20 students are held monthly at the University Hospital of North Staffordshire (UHNS). These sessions are led by a minimum of four peer tutors, who are clinical students in their third to fifth year of the 5-year medicine course at Keele. The peer tutors are recruited by the KSS and receive training by specialist registrars in surgery to ensure high-quality sessions are delivered.

Based on our personal involvement as: 1) a pre-clinical student attending the session; 2) a peer tutor leading the session; and 3) a session organizer, we present our experiences to illustrate the benefits and drawbacks of this type of PAL approach to teaching and learning basic surgical skills. Finally, we discuss potential implications of incorporating PAL sessions into a medical curriculum.

## The session organizer experience

As a session organizer, overseeing these surgical skills sessions was a challenging experience. Initially, there was some anxiety, as there were several ‘pieces’ that needed to smoothly come together (e.g., surgical equipment and competent peer tutors). Being a senior medical student was advantageous in that I could better understand other students’ motivations and learning needs. However, despite being only a couple of years ahead, and still a medical student, I was unsure of the response my fellow learners would have to a student-led session. Fortunately, the positive feedback I had received from the peer tutors and students suggested a PAL approach to these sessions was well-received.

Coordinating these sessions also helped me to develop my organizational and leadership skills. Session organizers had to advertise the session, recruit the correct number of students and peer tutors, procure and prepare the appropriate equipment and room, and develop teaching aids (e.g., videos, handouts) and feedback forms. The experience also helped me to adjust my level of information when explaining material to learners of different levels.

## The peer tutor experience

First, my role involved demonstrating basic suturing – including simple, interrupted suturing followed by square knot tying. Apart from demonstrating these surgical skills, I also made myself available throughout the session to assist anyone who was struggling. Initially, the thought of having to suture in front of my peers was a bit daunting. However, the combination of having to prepare myself before these sessions and then actively assist pre-clinical students positively reinforced my own abilities. Not only did I feel like my presentation skills improved, but I also strengthened relationships with my peers.

When registrars or consultants led these sessions, I found demonstrations were often rushed – perhaps because suturing is second nature and they fail to recognize that medical students need repetitive and exaggerated hand maneuvers to emphasize important steps. Also, registrars or consultants included more advanced suturing (e.g., subcuticular suture) that sometimes took precedence over the more basic procedures. We found this to be unnecessary, as a focus on mastering the basics provided the foundation for more advanced suturing.

## The pre-clinical student experience

As a pre-clinical student, attending a PAL session led by clinical year medical students with knowledge of basic surgical suturing and knot-tying techniques was new to me.

Having never attended a peer-led practical session before, I was initially apprehensive about how useful the session would be, and whether or not peers’ skills would be proficient enough to learn from. I was also worried that the learning curve would be too steep and challenging. Seeing some familiar faces helped to relax me – certainly more than I would have been in theatre or in a faculty-led teaching session. The informal environment and ample time allotted for the session helped me to really concentrate on learning the skills I was being taught.

Despite my initial uncertainties, tutors demonstrated reasonable background knowledge in suturing and knot tying to answer related questions. Even so, the honest advice given helped allay my anxiety and smooth my transition into the clinical environment.

## Conclusion

As these data represent only personal accounts of our various experiences, they may not reflect the general student population. Moreover, no formal evaluation was done to examine the effectiveness of a PAL approach relative to the traditional method of using registrars or consultants to teach basic surgical skills. In future, a much more rigorous attempt to better identify and understand the benefits and pitfalls of a ‘peer-on-peer’ approach would be well motivated.

Despite these obvious limitations, our experiences suggest that using PAL to teach basic surgical skills to pre-clinical students has much potential. Students who assume different roles within the PAL system appear to benefit and learn in ways that can be highly applicable to future endeavors (5). In turn, students who attend these sessions may benefit by learning an intricate yet crucial skill in a less stressful environment (Box 1).

Admittedly, peer tutors’ lack of training and experience in suturing is a concern that could potentially impact the attainment of related curricular learning objectives (6, 7). Thus, it is likely that some formal training for peer tutors, along with better communication between session organizers and medical school faculty/administrators, is needed before such sessions can be widely incorporated into medical curricula. Indeed, a combination of both PAL sessions and faculty-led teaching sessions may represent the optimal learning model for teaching basic, introductory surgical skills to pre-clinical students.

**Box 1. Possible benefits arising from each role within a PAL scheme in basic surgical skills**

Session organizer:

- Leadership skills
- Organizational skills
- Team-working and communication skills

Peer tutor:

- Presentation skills
- Communication skills
- Re-enforcement of suturing skills
- Improved confidence in basic surgical skills

Pre-clinical student:

- Opportunity to practice in an informal environment
- Observation of basic surgical skills being performed by peers
- Continuing advice and support from peers
- Improved confidence in basic surgical skills
